# Skin manifestations following anti‐COVID‐19 vaccination: A multicentricstudy from Turkey

**DOI:** 10.1111/jocd.15570

**Published:** 2022-12-27

**Authors:** Ilteris Oguz Topal, Aslı Tokmak, Gökçe Işıl Kurmuş, Göknur Kalkan, Düriye Deniz Demirseren, Mustafa Tosun, Selma Emre, Tuğba Özkök Akbulut, Hatice Kaya Özden, Mahmut Can Koska, Seray Külcü Çakmak, Ömer Kutlu, Emine Mutlu, Güneş Gür Aksoy, Filiz Topaloğlu Demir, Ayşe Serap Karadağ

**Affiliations:** ^1^ Department of Dermatology and Venereology, University of Health Sciences Prof. Dr. Cemil Tascioglu City Hospital Istanbul Turkey; ^2^ Department of Dermatology and Venereology Agri Training and Research Hospital Agri Turkey; ^3^ Department of Dermatology and Venereology, Faculty of Medicine Yüksek İhtisas University Ankara Turkey; ^4^ Department of Dermatology and Venereology Yildirim Beyazit University, Medical School Ankara Turkey; ^5^ Department of Dermatology and Venereology, University of Health Sciences Ankara City Hospital Ankara Turkey; ^6^ Department of Dermatology and Venereology Sivas Cumhuriyet University Sivas Turkey; ^7^ Department of Dermatology and Venereology, University of Health Sciences Haseki Training and Research Hospital Istanbul Turkey; ^8^ Department of Dermatology and Venereology Kocaeli Derince Training and Research Hospital Kocaeli Turkey; ^9^ Department of Dermatology and Venereology Artvin State Hospital Artvin Turkey; ^10^ Department of Dermatology and Venereology Gaziosmanpaşa University Medical Faculty Tokat Turkey; ^11^ Department of Dermatology and Venereology Cankiri State Hospital Cankiri Turkey; ^12^ Department of Dermatology and Venereology, Faculty of Medicine Istanbul Medipol University Istanbul Turkey; ^13^ Department of Dermatology and Venereology Memorial Ataşehir Hospital Istanbul Turkey

**Keywords:** COVID‐19, reactions, vaccine

## Abstract

**Purpose:**

After the emergence of the pandemic caused by the COVID‐19 virus, vaccination with various vaccines has started to be implemented across the world. To identify dermatological reactions developing after the COVID‐19 vaccines administered in Turkey and determine their clinical features and risk factors that may play a role in their development.

**Materials and Methods:**

The study included patients aged ≥18 years, who presented to 13 different dermatology clinics in Turkey between July 2021 and September 2021 after developing dermatological reactions following the administration of the COVID‐19 vaccine. After providing written consent, the patients were asked to complete a standard survey including questions related to age, gender, occupation, comorbidities, the regular medication used, the onset of cutaneous reactions after vaccination, and localization of reactions. Dermatological reactions were categorized according to whether they developed after the first or second dose of the vaccine or whether they occurred after the inactivated or messenger RNA (mRNA) vaccine. The relationship between dermatological reactions and some variables such as gender and comorbidities was also evaluated.

**Results:**

A total of 269 patients [116 women (43.1%), 153 men (56.9%)] were included in the study. It was observed that the dermatological diseases and reactions that most frequently developed after vaccination were urticaria (25.7%), herpes zoster (24.9%), maculopapular eruption (12.3%), and pityriasis rosea (4.5%). The rate of dermatological reactions was 60.6% after the administration of the mRNA vaccine and 39.4% after that of the inactivated vaccine. There was a statistically significantly higher number of reactions among the patients that received the mRNA vaccine (*p* = 0.001).

**Conclusion:**

The most common reactions in our sample were urticaria, herpes zoster, and maculopapular eruption. Physicians should know the dermatological side effects of COVID‐19 vaccines and their clinical features.

## 1 INTRODUCTION

In December 2019, a large number of unexplained and fatal pneumonia cases emerged in Wuhan. The disease caused by the SARS‐CoV‐2 virus, which is the agent isolated from the lower respiratory tract of infected patients, was named as coronavirus disease. The virus quickly spread across the world, causing a large number of deaths. In March 2020, the World Health Organization declared the disease a pandemic.^1^, ^2^

The coronavirus disease has a high rate of mortality, especially in elderly people and those with comorbidities. In order to prevent the spread of the virus and minimize associated negative effects, vaccine studies were started promptly. The results of the phase 3 study of the German‐origin Pfizer‐BioNTech messenger RNA (mRNA) vaccine were finally presented to the U.S. Food and Drug Administration, which granted emergency use permission for this vaccine to be used in healthcare workers and high‐risk patients in December 2020.^3^ For the inactivated Sinovac vaccine, phase 1/2 studies were carried out in China, and phase 3 studies were initiated in Brazil, Indonesia, Turkey, and China after receiving approval from the Chinese National Medical Products Administration to conduct human clinical trials in April 2020. In October, the inactivated vaccine was approved for use in high‐risk individuals.^4^ In Turkey, the Sinovac vaccine started to be administered as of January 2021 and the mRNA BioNTech vaccine as of March 2021.

With the implementation of COVID‐19 vaccines across the world, various dermatological diseases related to these vaccines, such as post‐vaccine urticaria, herpes zoster, radiation recall phenomenon, and Steven Johnson syndrome, have been reported.^5^, ^6^, ^7^, ^8^ New reports on the side effects of vaccines continue to be published every day, and it is expected that there will be more reports on this fairly new vaccine application in the coming period.

In this study, our aim was to determine dermatological reactions due to the COVID‐19 vaccines administered in Turkey, their onset and clinical features, and risk factors that may play a role in their development.

## 2 MATERIAL AND METHODS

This cross‐sectional observational study included patients aged ≥18 years, who presented to 13 different dermatology clinics in Turkey between July 2021 and September 2021 after developing dermatological reactions following the administration of the COVID‐19 vaccine. After providing informed consent, the patients were asked to complete a standard survey including questions related to age, gender, occupation, comorbidities, regular medications used, onset of cutaneous symptoms after vaccination, localization of reactions, type of vaccine administered applied, whether there was any symptom, whether they experienced a similar health problem before, and they had a history of COVID‐19. The patients were followed up, and for each patient, it was noted how many days it took for cutaneous symptoms to regress.

The frequency and type of dermatological reactions were evaluated according to gender, age being ≥50 or < 50 years, presence of comorbidities, presence of regular medication use, presence of allergic dermatological diseases, and body mass index (BMI) being ≥30 or < 30. Patients who were infected with COVID‐19 despite receiving the COVID‐19 vaccine, those with an active infection, and those under the age of 18 years were excluded from the study. The study was approved by the medical ethics committee of the Clinical Ethics Committee (approval number: 24/06/2021–201).

### 2.1 Statistical analysis

SPSS v. 15.0 for Windows was used for statistical analyses. Descriptive statistics were presented as numbers and percentages for categorical variables, and mean, standard deviation, minimum, maximum, and median values for numerical variables. Rates in independent groups were compared with the chi‐square test. The statistical alpha significance level was accepted as *p* ≤ 0.05.

## 3 RESULTS

A total of 269 patients (116 women [43.1%], 153 men [56.9%]) were included in the study. The mean age was 50 ± 1.0 (18–91) years. The demographic characteristics of the patients are summarized in Table 1.

**TABLE 1:** Demographic and clinical characteristics of the patients.

Variable		Mean ± Standard Error (Min‐Max)	Number	Percentage
Age (years)		50 ± 1.0 (18–91)	269	
Age group	<19 years, young		4	1.5
	19–35 years, young adult		54	20.1
	36–65 years, adult		144	53.5
	>65 years, elderly		67	24.9
Gender	Female		116	43.1
	Male		153	56.9
Obesity	Absent, BMI < 30		217	80.7
	Present, BMI ≥30		52	19.3
Smoking status	Non‐smoker		208	77.3
	Smoker		61	22.7
Alcohol consumption	Absent		249	92.6
	Present		20	7.4
Allergic disease	Absent		216	80.3
	Present		53	19.7
Egg allergy	Absent		266	98.9
	Present		3	1.1
Type of allergic disease	Atopic dermatitis		6	9.5
	Asthma		15	23.8
	Allergic rhinitis		18	28.6
	Allergic conjunctivitis		6	9.5
	Drug allergy		10	15.9
	Urticaria		7	11.1
	Other		1	1.6
	Total		63	100.0
History of dermatological disease	Absent		222	82.5
	Present		47	17.5
Previous vaccine reaction	Absent		261	97.0
	Present		8	3.0
Type and dose of vaccine administered	First Sinovac dose		42	15.6
	Second Sinovac dose		64	23.8
	First BioNTech dose		125	46.5
	Second BioNTech dose		38	14.1
	Total		269	100.0
Dermatological reaction at first dose	Absent		112	41.6
	Present		36	13.4
	Not known		121	45.0
Similar previous complaint	Absent		236	87.7
	Present		33	12.3

**FIGURE 1:**
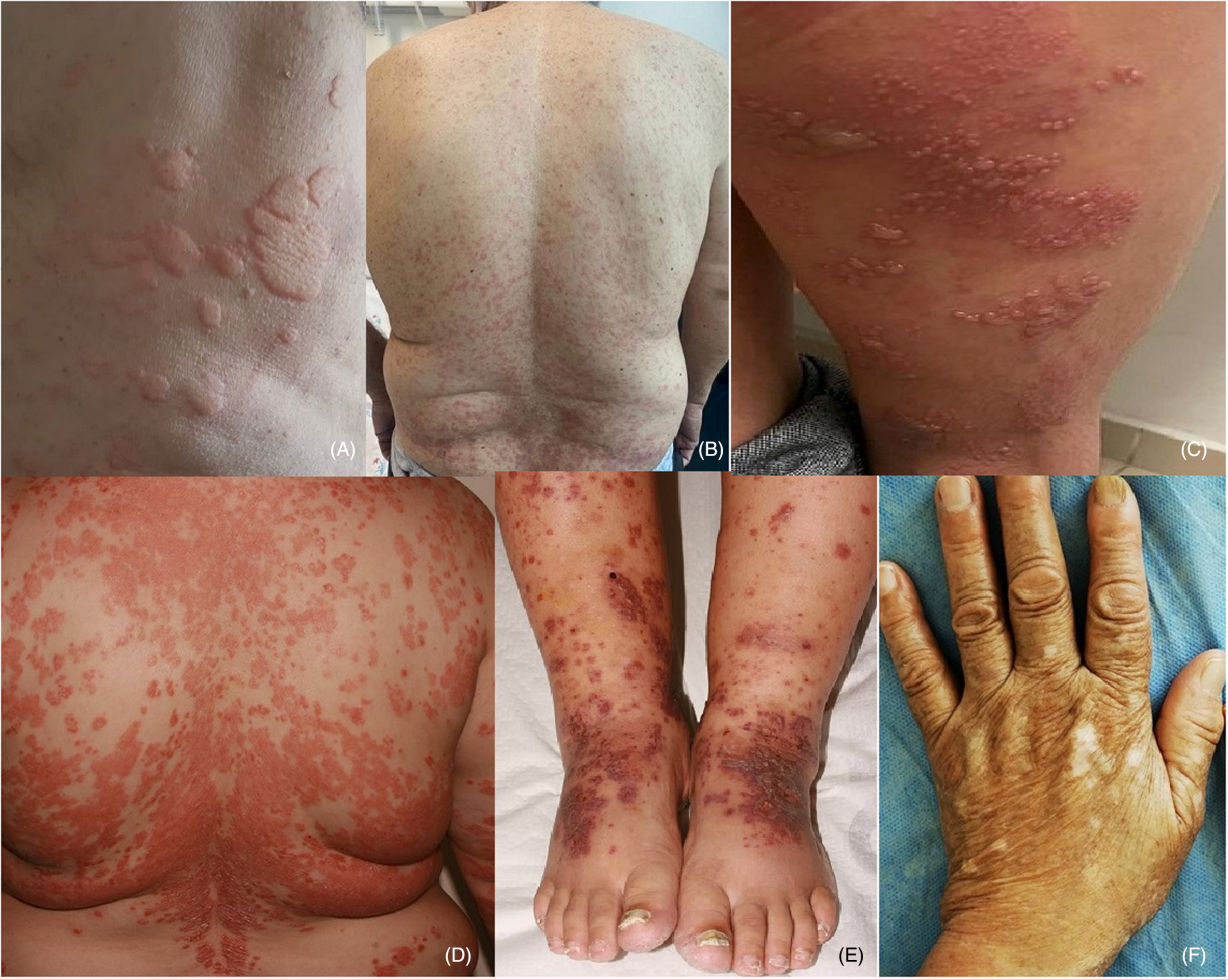
Some dermatological reactions after administration of BioNTech/Pfizer or inactivated vaccine. (A) urticaria, (B) maculopapular eruption, (C) herpes zoster, (D) psoriasis, (E) leukocytoclastic vasculitis, and (F) vitiligo

The dermatological diseases and reactions that most frequently developed after vaccination were urticaria (25.7%), herpes zoster (24.9%), maculopapular eruption (12.3%), and pityriasis rosea (4.5%) (Figure 1). Other dermatological diagnoses are also shown in Table 2.

**TABLE 2:** Distribution of the dermatological reactions of the patients by gender and age.

Dermatological diagnosis	Total		Gender					Age Group				
			Female		Male		*p*	<=50 years		>50 years		*p*
	*n*	%	*n*	%	*n*	%		*n*	%	*n*	%	
**Urticaria**	69	25.7	30	11.2	39	14.5	0.945	48	17.8	21	7.8	**<0.001**
**Herpes zoster**	67	24.9	32	11.9	35	13.0	0.376	16	5.9	51	19.0	**<0.001**
**Maculopapular eruption**	33	12.3	16	5.9	17	6.3	0.634	19	7.1	14	5.2	0.471
**Pityriasis rosea**	12	4.5	5	1.9	7	2.6	1.000	6	2.2	6	2.2	1.000
**Vasculitis**	10	3.7	3	1.1	7	2.6	0.522	4	1.5	6	2.2	0.540
**Psoriasis**	10	3.7	5	1.9	5	1.9	0.750	8	3.0	2	0.7	0.103
**Erythema multiforme**	6	2.2	1	0.4	5	1.9	0.240	5	1.9	1	0.4	0.214
**Angioedema**	6	2.2	3	1.1	3	1.1	1.000	4	1.5	2	0.7	0.684
**Bullous pemfigoid**	6	2.2	4	1.5	2	0.7	0.408	0	0.0	6	2.2	**0.014**
**Injection site reaction**	6	2.2	1	0.4	5	1.9	0.240	4	1.5	2	0.7	0.684
**Vitiligo**	6	2.2	3	1.1	3	1.1	1.000	3	1.1	3	1.1	1.000
**Fixed drug reaction**	5	1.9	3	1.1	2	0.7	0.655	4	1.5	1	0.4	0.370
**Pruritus**	5	1.9	0	0.0	5	1.9	0.072	0	0.0	5	1.9	**0.030**
**Petechiae**	3	1.1	2	0.7	1	0.4	*p* > 0.05	1	0.4	2	0.7	*p* > 0.05
**Erythema nodosum**	3	1.1	2	0.7	1	0.4	*p* > 0.05	2	0.7	1	0.4	*p* > 0.05
**Bacterial skin infection**	3	1.1	3	1.1	0	0.0	*p* > 0.05	3	1.1	0	0.0	*p* > 0.05
**Herpes simplex**	3	1.1	0	0.0	3	1.1	*p* > 0.05	2	0.7	1	0.4	*p* > 0.05
**Lichenoid drug reaction**	2	0.7	1	0.4	1	0.4	*p* > 0.05	2	0.7	0	0.0	*p* > 0.05
**Other†**	14	5.60	2	0.8	12	4.8	‐	4	1.6	10	4	‐
**Total**	269	100.0	116	43.1	153	56.9		135	50.2	134	49.8	

*Note*: †Includes lymphadenopathy (*n* = 1), drug rash with eosinophilia and systemic symptoms syndrome (*n* = 1), pityriasis lichenoides chronica (*n* = 1), pseudolymphoma (*n* = 1), oral candidiasis (*n* = 1), telogen effluvium (*n* = 1), hyperhidrosis (*n* = 1), nummular dermatitis (*n* = 1), erythema intertrigo (*n* = 1), photoallergic contact dermatitis (*n* = 1), onycholysis (*n* = 1), and hypopigmentation (*n* = 1) in men. Perforating collagenosis (n = 1), edema in the flap area (n = 1) in women.

Statistically significant (*p* < 0.05) for bold values.

**FIGURE 2:**
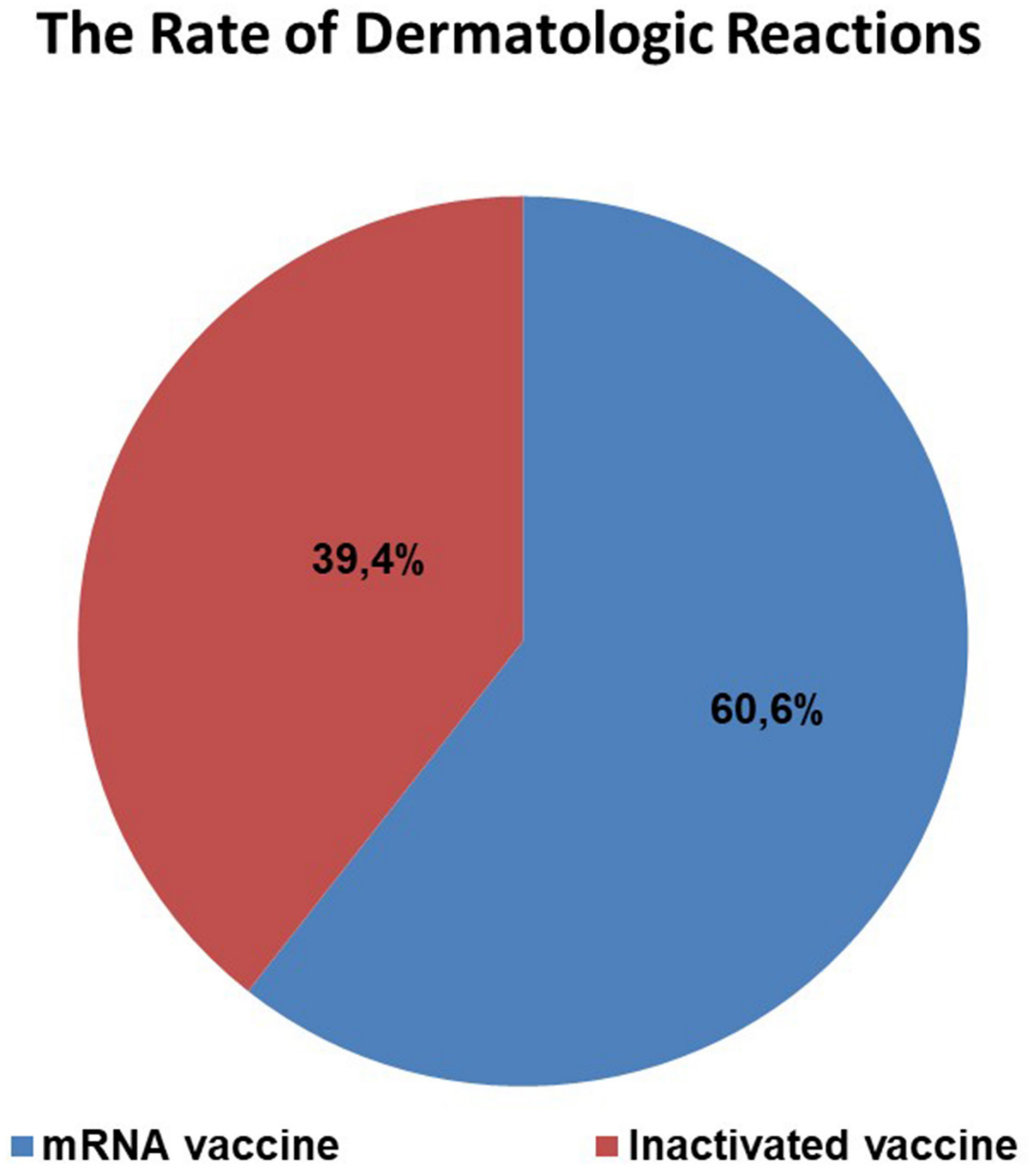
Rate of dermatological reactions by the type of vaccine

The rate of dermatological reactions was determined as 60.6% after the mRNA vaccine and 39.4% after the inactivated vaccine. There was a statistically significant higher number of reactions among the patients that received the mRNA vaccine (*p* = 0.001) (Figure 2).

The first three most common dermatological reactions after the inactivated vaccine were herpes zoster (*n* = 39; 14.5%), urticaria (*n* = 18; 6.69%), and maculopapular eruption (*n* = 13; 4.84%). The three most common dermatological reactions after the mRNA vaccine were urticaria (*n* = 51; 18.96%), herpes zoster (*n* = 28; 10.41%), and maculopapular eruption (*n* = 20; 7.44%). All the injection site reactions were seen after the mRNA vaccine (2.23%). Bullous pemphigoid (BP) development was more common in the inactivated vaccine group than in the mRNA vaccine group (1.86% and 0.37%, respectively). Other dermatological reactions are shown in Table 3.

**TABLE 3:** Distribution of dermatological reactions by the type and dose of vaccine.

Dermatological reaction	First BioNTech dose (***n*** = 125) ***n*** (%)	Second BioNTech dose (***n*** = 38) ***n*** (%)	First Sinovac dose (***n*** = 42) ***n*** (%)	Second Sinovac dose (***n*** = 64) ***n*** (%)
**Urticaria**	38 (14.13)	13 (4.83)	11 (4.09)	7 (2.6)
**Herpes zoster**	21 (7.81)	7 (2.6)	17 (6.32)	22 (8.18)
**Maculopapular eruption**	17 (6.32)	3 (1.12)	4 (1.49)	9 (3.35)
**Vasculitis**	8 (2.97)	‐	1 (0.37)	1 (0.37)
**Psoriasis**	4 (1.49)	4 (1.49)	1 (0.37)	1 (0.37)
**Injection site reaction**	5 (1.86)	1 (0.37)	‐	‐
**Pityriasis rosea**	3 (1.12)	2 (0.74)	2 (0.74)	5 (1.86)
**Erythema multiforme**	5 (1.86)	‐	‐	1 (0.37)
**Vitiligo**	4 (1.49)	1 (0.37)	‐	1 (0.37)
**Angioedema**	3 (1.12)	1 (0.37)	‐	2 (0.74)
**Fixed drug reaction**	4 (1.49)	‐	1 (0.37)	‐
**Petechiae**	2 (0.74)	‐	‐	1 (0.37)
**Erythema nodosum**	3 (1.12)	‐	‐	‐
**Bullous pemphigoid**	‐	1 (0.37)	2 (0.74)	3 (1.12)
**Herpes simplex**	1 (0.37)	1 (0.37)	1 (0.37)	‐
**Bacterial skin infection**	1 (0.37)	‐	‐	2 (0.74)
**Pruritus**	‐	1 (0.37)	‐	4 (1.49)
**Other †**	6 (2.22)	3 (1.11)	2 (0.74)	5 (1.85)

*Note*: †Includes lymphadenopathy (*n* = 1), lichenoid drug reaction (*n* = 1), pityriasis lichenoides chronica (*n* = 1), pseudolymphoma (*n* = 1), oral candidiasis (*n* = 1), telogen effluvium (*n* = 1), photoallergic contact dermatitis (*n* = 1), hyperhidrosis (*n* = 1), edema in the flap area (*n* = 1) in the BioNTech group. Hypopigmentation (*n* = 1), drug rash with eosinophilia and systemic symptoms syndrome (*n* = 1), lichenoid drug reaction (*n* = 1), nummular dermatitis (n = 1), onycholysis (*n* = 1), erythema intertrigo (*n* = 1), perforating collagenosis (*n* = 1) in the Sinovac group.

At the time of presentation, the rate of patients who had received the first dose of BioNTech was 46.5%, while the rate of those who had received the second dose of the BioNTech vaccine was 14.1%. For the Sinovac vaccine, these rates were 15.6 and 23.8, respectively (Table 1).

Dermatological reactions developed within the first 12 h of vaccination in 19 patients (7.1%), between 12 and 24 h in 36 patients (13.4%), and between one and 3 days in 60 patients (22.3%). In terms of onset, dermatological reactions mostly developed between the third and seventh days (*n* = 78; 29%). The onset times of dermatological reactions are summarized in Table 4.

**TABLE 4:** Distribution of dermatological reactions by onset time or regression time and type of vaccine.

Onset time		Type of vaccine									
		First Sinovac dose		Second Sinovac dose		First BioNTech dose		Second BioNTech dose		Total	
		*N*	%	*N*	Table %	*N*	Table %	*N*	Table %	*N*	Table %
Onset time of dermatological reactions Mean ± SD (Min‐Max) 7 ± 0.5 (1–60) days	<12 h	2	0.74	1	0.37	15	5.58	1	0.37	19	7.06
	>12 hours‐1 day	3	1.12	5	1.86	23	8.55	5	1.86	36	13.38
	1–3 days	11	4.09	11	4.09	29	10.78	9	3.35	60	22.30
	3–7 days	17	6.32	18	6.69	31	11.52	12	4.46	78	29.00
	7–14 days	4	1.49	18	6.69	12	4.46	6	2.23	40	14.87
	14–21 days	4	1.49	7	2.60	12	4.46	3	1.12	26	9.67
	21–28 days	1	0.37	3	1.12	1	0.37	1	0.37	6	2.23
	>28 days	0	0.00	1	0.37	2	0.74	1	0.37	4	1.49
	Total	42	15.61	64	23.79	125	46.47	38	14.13	269	100.00
Regression of the reaction	Yes	40	14.98	58	21.72	102	38.2	27	10.11	227	85.02
	No	2	0.75	6	2.25	21	7.87	11	4.12	40	14.98
	Total	42	15.73	64	23.97	123	46.07	38	14.23	267	100.00
Regression time of the reactions Mean ± SD (Min‐Max) 12 ± 0.8 (1–90) days	2–3 days	5	2.20	4	1.76	18	7.93	4	1.76	31	13.66
	3–7 days	14	6.17	13	5.73	39	17.18	3	1.32	69	30.40
	7–14 days	13	5.73	20	8.81	18	7.93	10	4.41	61	26.87
	14–21 days	1	0.44	12	5.29	10	4.41	5	2.20	28	12.33
	21–28 days	0	0.00	0	0.00	1	0.44	0	0.00	1	0.44
	>28 days	2	0.88	7	3.08	6	2.64	5	2.20	20	8.81
	Total	40	17.62	58	25.55	102	44.93	27	11.89	227	100.00

The number of symptomatic patients was 267 (99.26%), and the three most common symptoms were itching, pain, and burning sensation. Antihistamine and topical steroid treatments were sufficient in most cases. A considerable percentage of dermatological reactions (30.4%) regressed in three to 7 days (Table 4).

In the analyses performed to determine factors that played a role in the development of dermatological reactions following vaccination, the rate of these reactions was found to be statistically significantly higher in the men (56.9%) than in the women (43.1%) (*p* = 0.02).

There was no significant difference between the <50 and ≥ 50 years groups in terms of the development rates of dermatological reactions (50.2% and 49.8%, respectively; *p* > 0.5).

While urticaria was more commonly seen in <50 years group than ≥50 years group (*p* < 0.001), herpes zoster, bullous pemfigoid, and pruritus were more commonly seen in ≥50 years group than <50 years group (*p* < 0.001) according to statistical analysis.

The detailed age analysis revealed that the rate of reaction development was higher in the 36–65 years group compared to the remaining age groups (*p* < 0.001). The patients with a BMI of ≥30 had a statistically significantly lower rate of dermatological reactions compared to those with a BMI of <30 (19.33% and 80.67%; *p* < 0.001). The rate of dermatological reactions was statistically significantly lower in the patients with no allergic disease (79.18%) compared to those with an allergic disease (20.82%) and in the patients with no dermatological disease (82.1%) compared to those with a dermatological disease (17.84%) (*p* < 0.001 for both).

## 4 DISCUSSION

It has been observed that urticaria, redness, edema, and injection site reactions may occur in phase 3 studies on the subcutaneous side effects of COVID‐19 vaccine.^9^, ^10^

In the current study, the patients most developed urticaria (*n* = 69; 25.7%) associated with the COVID‐19 vaccines. In a study screening 414 individuals who had been vaccinated against COVID‐19, it was observed that urticaria developed in 40 patients (9.6%). It was stated that most of these patients developed urticaria after the first dose.^11^ Similarly, in our study, urticaria was most observed after the first dose of vaccination (18.2%). In another study evaluating cutaneous side effects of the Pfizer–BioNTech vaccine among healthcare workers in the Czech Republic, the rate of those that developed urticaria was reported to be 22.2%,^12^ which is in agreement with our findings (Table 5).

**TABLE 5:** Comparison with current study and the other studies in terms of the rate of skin reactions associated with BioNTech vaccine.

Diagnosis	Current study N (%)	McMahon DE et al. *N* (%)	Riad A et al. *N*(%)	El‐Shitany NA et al. *N*(%)	Català A et al. *N*(%)	Fernandez‐Nieto D et al. *N*(%)
Urticaria	51(18.96)	17 (22.9)	10 (22.2)	‐	24 (14.7) (with or without angioedema)	2 (0.04)
Herpes zoster	28 (10.41)	5 (6.7)	‐	‐	28 (17.2)	‐
Maculopapular eruption	20 (7.44)	9 (12.1)	28 (62.2)	‐	19 (11.7)	‐
Vasculitis	8 (2.97)	1 (1.35)	‐	‐	‐	‐
Injection site reaction	6(2.23)	18 (24.3)	731 (89.3)	60 (63.8)	23 (14.1)	103 (2.1)
Psoriasis	8 (2.98)	‐	‐	‐	‐	‐
Pityriasis rosea	5 (1.86)	3 (4)	‐	‐	11(6.7)	
Angioedema	4 (1.49)	1 (1.35)	‐	‐	‐	‐
Erythema multiforme	5 (1.86)	0	‐	‐	‐	‐
Bullous pemphigoid	1 (0.37)	‐	‐	‐	‐	‐
Vitiligo	5 (1.86)	‐	‐	‐	‐	‐
Herpes simplex	2 (0.74)	‐	‐	‐	5 (3.1)	
Pruritus	1 (0.37)	‐	‐	‐	‐	70 (68)

Post‐vaccine severe allergic reactions and anaphylaxis are very rare, with a rate of 0.9–1.8 (95% confidence interval) in 1.31 million doses.^13^, ^14^ Anaphylaxis was reported in 21 patients at a rate of 11.1 per million doses after the Pfizer‐BioNTech COVID‐19 vaccine.^15^ Angioedema developed in six of our patients (2.2%), and it was found to develop within minutes of the administration of the vaccine in three (1.1%) of these patients, which is consistent with the literature.

In our study, herpes zoster was the second most common cutaneous reaction in the whole sample (24.9%). When the vaccine groups were groups, the rate of herpes zoster was significantly higher in the inactivated vaccine than in BioNTech (14.5% and 10.41%, respectively). In the literature, it has been reported that herpes virus reactivation occurs after influenza, hepatitis A, and rabies vaccines.^16^ It has been considered that immunomodulation, which includes alloreactivity caused by inactivated hepatitis B vaccines and suppression of cellular immunity through live vaccines, can cause herpes virus reactivation.^17^ Therefore, it is suggested that immunodysregulation after COVID‐19 vaccination may result in herpes zoster reactivation.^18^ In a study, the authors observed that herpes zoster developed in 10% of patients after the second dose of the Pfizer‐BioNTech vaccine.^11^ In another study conducted in Spain, herpes zoster and herpes simplex reactivation was reported at a rate of 13.8%.^19^ In the same study, the most common cutaneous side effect of the Pfizer‐BioNTech, was determined as herpes zoster reactivation at a rate of (17.2%). This finding was compatible with our study's result.

In our study, the rate of patients who developed pityriasis rosea was 4.5% (*n* = 12). We had five patients (1.85%) who developed new psoriasis and a further five cases with psoriasis exacerbation. Cases of pityriasis rosea associated with COVID‐19 vaccines were recently reported. It was suggested that the vaccine might cause HHV‐6 and HHV‐7 reactivation or create the disease setting by mimicking viral epitopes and triggering a T‐cell response.^20^ There are also reports on the emergence of psoriasis, another erythematous‐scaly disease, or the exacerbation of existing psoriasis.^21^, ^22^, ^23^ In a recent study evaluating 419 cutaneous reactions due to COVID‐19 vaccines, the rate of patients developing pityriasis rosea was reported to be 4.9% (*n* = 20). In the same study, the rate of patients with the exacerbation of existing psoriasis was 1.4% (*n* = 6), and the rate of those with newly developed psoriasis was 0.7% (*n* = 3).^19^

There are case reports on the development of erythematous rash after the first or second dose of COVID‐19 vaccines.^24^, ^25^ McMahon et al.'s study, 27 (6.5%) individuals reported the development of morbilliform eruption. Nine of these patients (2.1%) developed this reaction after the Pfizer‐BioNTech vaccine.^11^ In another study, Riad et al. found rash to be the most common cutaneous side effect (62.2%) related to the BioNTech vaccine^12^ (Table 5).

In a single‐center study, the side effects of the Pfizer‐BioNTech vaccine were evaluated in 3170 healthcare workers. Cutaneous symptoms, such as erythema, edema, diffuse morbilliform eruption, and urticaria, were observed in 38% of the patients (*n* = 11).^26^ In this current study, maculopapular eruption was found in 12.3% of the patients, of whom 7.44% (*n* = 20) developed this reaction after the Pfizer‐BioNTech vaccine.

Leukocytoclastic vasculitis has also been reported in some patients due to COVID‐19 vaccines. It is considered that vaccine proteins similar to SARS‐CoV‐2 antigens may induce vasculitis by causing an autoreactive T/B‐cell response, antibody production, and immunocomplex deposition.^27^, ^28^ McMahon et al.'s study, the rate of patients with vasculitis was 3.6%.^11^ Similarly, in our study, 3.7% of the patients developed vasculitis. While one of these patients had urticarial vasculitis, the others had leukocytoclastic vasculitis.

In a study evaluating subepidermal bullous eruptions following vaccination, a total of eight patients were diagnosed with BP. The question of whether this development is a coincidence has been a matter of curiosity; however, it is also known that BP cases have been previously reported after measles, shingles, influenza, and hepatitis B vaccines.^29^ It was considered that the vaccine triggered the production of antibodies.^30^ In our study, BP occurred in six patients (2.2%).

Recently, a 58‐year‐old male patient with ulcerative colitis was reported to develop vitiligo after the first dose of the Pfizer/BioNTech.^31^ In our study, we found that six (2.2%) patients had biopsy‐confirmed vitiligo development. The effects of cytokines, such as IL‐6, interferon gamma (IFN‐γ), and tumor necrosis factor‐alpha, have been previously described in the pathogenesis of vitiligo.^32^ Therefore, it seems likely that vitiligo is triggered by the release of IFN‐γ, which play a role in vaccine response.

When we evaluated our patients in terms of age, the patients aged <50 years and those aged ≥50 years were compared, there was no significant difference in dermatological reactions (50.2% and 49.8%, respectively). However, the rate of reaction development was statistically significantly higher in adults aged 36–65 years compared to the remaining age groups (*p* < 0.001). In a Czech study, the rates of cutaneous side effects associated with the BioNTech vaccine were found to be 6.2% in patients aged ≤43 years and 4.1% in those aged >43 years. In the analysis of age groups, it was observed that there was no statistically significant difference between these two age groups. In the same study, it was noted that cutaneous side effects were mostly seen in the upper extremities (60%).^12^ In our patients, dermatological reactions most frequently occurred on the anterior aspect of the trunk (24%).

Most of our patients (99.2%) were symptomatic, with the most common symptoms being identified as itching, pain, and burning sensation. We found that vaccine‐related dermatological reactions mostly occurred within the first 7 days. In the subsequent follow‐up of the patients, we observed that the symptoms and signs regressed in up to 14 days in most of the patients. In a Spanish study, it was determined that the patients had symptoms such as itching, pain, and burning and pricking sensations at various rates.^19^

In the current study, cutaneous side effects were observed in 163 of the patients that had been administered the mRNA vaccine and 106 of those that had received the inactivated vaccine. The rate of dermatological side effects was statistically significantly higher in the mRNA vaccine group than in the inactivated vaccine group (*p* = 0.001).

The limitations of our study include the inability to perform a statistical analysis due to the small number of patients in some subgroups and the absence of a control group.

The number of COVID‐19 vaccines administered in the world is increasing day by day. It is expected that studies conducted by health ministries in various countries increased awareness of the public on this issue and widespread application of vaccines will result in an increase in the detected incidence of dermatological complications. Therefore, it is important for physicians to know the dermatological side effects of vaccines and their clinical features, and develop treatment strategies accordingly. We consider that current literature information will increase with new studies on vaccines and cutaneous findings reported from different centers.

## AUTHOR CONTRIBUTIONS

Ilteris Oguz Topal and Ayşe Serap Karadağ involved in conception, critical review, writing, supervision, and design. Ilteris Oguz Topal, Aslı Tokmak, Gökçe Işıl Kurmuş, Göknur Kalkan, Düriye Deniz Demirseren, Mustafa Tosun, Selma Emre, Tuğba Özkök Akbulut, Hatice Kaya Özden, Mahmut Can Koska, Seray Külcü Çakmak, Ömer Kutlu, Emine Mutlu, Güneş Gür Aksoy, Filiz Topaloğlu Demir, and Ayşe Serap Karadağ involved in data collection and/or processing. Ilteris Oguz Topal involved in literature review.

## CONFLICT OF INTEREST

None.

## ETHICAL APPROVAL

This study was approved by the Ethics Committee of the Prof.Dr.Cemil Tascioglu City Hospital (Istanbul, Turkey) (approval number: 24/06/2021‐201).

## FINANCIAL SUPPORT

None.

## Data Availability

The data that support the findings of this study are openly available in [repository name] at [DOI].
